# Trehalose Protects against Superoxide Dismutase 1 Proteinopathy in an Amyotrophic Lateral Sclerosis Model

**DOI:** 10.3390/antiox13070807

**Published:** 2024-07-03

**Authors:** Rayne S. S. Magalhães, José R. Monteiro Neto, Gabriela D. Ribeiro, Luan H. Paranhos, Elis C. A. Eleutherio

**Affiliations:** Institute of Chemistry, Federal University of Rio de Janeiro (UFRJ), Rio de Janeiro 21941-901, Brazil; rayne.stfhany@gmail.com (R.S.S.M.); jraphamonteiro0810@gmail.com (J.R.M.N.); gdelaqua@gmail.com (G.D.R.); luan.holanda@outlook.com (L.H.P.)

**Keywords:** trehalose, amyotrophic lateral sclerosis, superoxide dismutase 1, oxidative stress

## Abstract

This work aimed to study the effect of trehalose in protecting cells against Sod1 proteinopathy associated with amyotrophic lateral sclerosis (ALS). Humanized yeast cells in which native Sod1 was replaced by wild-type human Sod1 or an ALS mutant (WT-A4V Sod1 heterodimer) were used as the experimental model. Cells were treated with 10% trehalose (p/v) before or after the appearance of hSod1 proteinopathy induced by oxidative stress. In both conditions, trehalose reduced the number of cells with Sod1 inclusions, increased Sod1 activity, and decreased the levels of intracellular oxidation, demonstrating that trehalose avoids Sod1 misfolding and loss of function in response to oxidative stress. The survival rates of ALS Sod1 cells stressed in the presence of trehalose were 60% higher than in their absence. Treatment with trehalose after the appearance of Sod1 inclusions in cells expressing WT Sod1 doubled longevity; after 5 days, non-treated cells did not survive, but 15% of cells treated with sugar were still alive. Altogether, our results emphasize the potential of trehalose as a novel therapy, which might be applied preventively in ALS patients with a family history of the disease or after diagnosis in ALS patients who discover the disease following the first symptoms.

## 1. Introduction

Amyotrophic lateral sclerosis (ALS) is a progressive and devastating aging-associated disorder affecting 4 to 5 individuals in 100,000 and is more prevalent in people between 55 and 75 years of age [1]. ALS targets motor neurons, and death often results from respiratory failure within 3–5 years after the first symptoms. The cost for treatment and care of patients is very expensive. Treatment is palliative, and the medications riluzole, edaravone, and relyvrio (sodium phenylbutyrate and taurursodiol), approved by the Food and Drug Administration (FDA) and other regulatory agencies, slow progression and extend survival by a few months [2]. Most ALS cases (90%) are sporadic (sALS) with no defined genetic origin, and only the minority show a familial predisposition (fALS) with Mendelian or non-Mendelian patterns of inheritance [3]. An important pathological hallmark of ALS is the accumulation of misfolded proteins in affected neurons and associated glial cells [4].

One of the most frequent forms of fALS is associated with mutations in the *SOD1* gene encoding the essential antioxidant enzyme Cu/Zn superoxide dismutase, leading to alterations in the folding and function of the protein [5]. Importantly, the wild-type (WT) Sod1 protein, present in sALS, can also misfold and form oligomers, suggesting that this is a central problem in ALS [6]. Sod1 is crucial to neuronal metabolism and health: (i) it is an important antioxidant enzyme of neurons, which are deficient in catalase and glutathione; (ii) Sod1 activates the expression of genes, which are important to protect neurons against oxidative stress; and (iii) it also regulates the shift from oxidative to fermentative metabolism, which is important for astrocyte-neuron metabolic cooperation reviewed in [7]. 

In our studies, we used simple experimental models, such as yeast *Saccharomyces cerevisiae* cells, to better understand the context of protein aggregation in the disease [8,9]. We used a strategy that mimics the genetic context of fALS, where most patients carry one copy of the WT *SOD1* gene and one copy carrying a genetic alteration. In patients, we think that the presence of a mutant copy of Sod1 alters the behavior of the WT copy. By taking advantage of powerful molecular imaging approaches, which enable the direct visualization of protein complexes in the cell (a technique known as bimolecular fluorescence complementation, BiFC), we were able to detect ‘hetero complexes’ formed by WT and the mutant human (h) Sod1 protein, which is a dimer. We found that yeast cells expressing hSod1 heterodimers showed decreased antioxidant activity, increased oxidative damage, reduced longevity, and oxidative stress-induced hSod1 aggregation [8,9]. Our group demonstrated clearly that aging associated with increased levels of oxidative stress induces the formation of hSod1 inclusions containing WT and/or mutant forms of Sod1.

Multiple compounds with reported effects on Sod1 function and aggregation are currently underway in clinical trials for Sod1-linked fALS and sALS [10]. These therapies aim to reduce Sod1 toxicity by either restoring the physiological Sod1 structure and role or by reducing the total amount of the Sod1 protein available to misfold and assemble. The latest strategy, recently approved in the U.S., the antisense oligonucleotide tofersen, which reduces Sod1 synthesis, is controversial because the removal of the Sod1 protein may predispose neurons to degeneration [11]. Therefore, therapies able to avoid Sod1 unfolding and aggregation and, consequently, to maintain activity have great potential to offer benefits to all ALS patients [6]. Trehalose, a non-reducing sugar of rare configuration (two molecules of glucose linked at their anomeric carbons), is a good candidate to perform this task. The FDA and other regulatory agencies issued a generally recognized as safe (GRAS) status for trehalose, a naturally occurring disaccharide found in bacteria, fungi, plants, insects, and invertebrates [12]. In vitro and in vivo studies have illustrated the extraordinary property of this molecule in protecting biological membranes and proteins during adverse conditions, such as oxidative stress [13]. It can stabilize the native conformation of proteins and avoid the aggregation of denatured proteins [14]. The physical and chemical properties of trehalose arise from the 1,1-glycosidic linkage, which leads to chemical stability and high hydrophilicity [15].

The therapeutic potential of trehalose in neurodegenerative diseases is based on its capacity to reduce the amount of reactive oxygen species and oxidative damage, reduce inflammatory responses, and decrease protein pathology by acting as a chemical chaperone, affecting protein folding and aggregation [16]. Trehalose treatment was reported to have modest neuroprotective effects in transgenic animal models carrying mouse G86R Sod1, which is considerably less aggregation-prone than human G85R Sod1 [17,18]. In addition, when given orally to the mouse model expressing human G93A Sod1, trehalose failed to slow down the disease progression, which has led to questions about the uptake and distribution of the molecule in this mouse strain [19]. Mammals, including humans, do not synthesize trehalose but are able to degrade trehalose through a specific enzyme, trehalase, present in the kidney, intestine, and liver. Therefore, the strategy to deliver trehalose to ALS models is very important for the success of this treatment. It is also important to use models that can simulate both sALS and Sod1-linked fALS, especially the most aggressive and common mutations, such as the heterozygous WT-A4V *SOD1* [3,9].

This work aimed to investigate the effect of trehalose on the survival extension of a humanized yeast model in which the endogenous yeast Sod1 was replaced by human Sod1, WT homodimer, or WT-A4V heterodimer. Trehalose addition occurred before or after the appearance of Sod1 inclusions, which were produced along chronological aging to verify the capacity of trehalose to prevent and/or reduce proteotoxicity. In order to better evaluate the effect of trehalose on proteotoxicity, Sod1 aggregation, Sod1 activity and expression, and the level of oxidative stress were also evaluated.

## 2. Material and Methods

### 2.1. Yeast Strains and Growth Conditions

The cells of the *sod1*Δ yeast strain (*MATa*; *his3*, *leu2*, *met15*; *ura3*; *sod1*::*KanMX4*), acquired from Euroscarf were transformed with hSOD1-BiFC plasmids, as previously described [9]. Those plasmids contain either one copy of WT human (h) *SOD1* cDNA fused to the sequence of the N-terminal part of the Venus fluorescent protein (VN) or one copy of WT *hSOD1* fused to the complementary sequence of the C-terminal part of Venus (VC), both under the control of the yeast *SOD1* promoter (SOD1WT strain). To construct the heterodimer mutant (SOD1WT-A4V strain), yeast cells were transformed with the plasmids containing WT hSOD1–VN and A4V hSOD1 mutant–VC. Cells were grown at 28 °C and 160 rpm up to the mid-exponential phase in a dropout 2% glucose medium without leucine and uracil in flasks 1/5 full.

### 2.2. Induction of Oxidative Stress and Treatment with Trehalose

Two different approaches were used to analyze the effect of trehalose to protect cells against hSod1 proteinopathy induced by aging-triggered oxidative stress [20]. To test the capacity of trehalose to prevent hSod1 proteinopathy in response to aging (Supplementary Material, Figure S1), cells growing on glucose (non-stressed cells, control condition) were harvested by centrifugation, washed twice with sterile-distilled water, and resuspended in the same volume of water, either without (W/O) or containing 10% p/v trehalose (T). Cells were incubated at 37 °C/160 rpm for 24 h (aged cells, condition A). To increase stress severity, part of the glucose-growing cell culture was treated with 0.4 mM of H_2_O_2_ at 28 °C/160 rpm for 1 h before being transferred to water, without (W/O) or with trehalose (T) (aged cells previously stressed with peroxide, condition H_2_O_2_ + A). To verify the ability of trehalose to reduce hSod1 proteinopathy (Supplementary Material, Figure S2), cells were treated with trehalose after 24 h of aging at 37 °C/160 rpm, with (WT hSOD1 cells—condition H_2_O_2_ + A) or without (WT-A4V hSOD1 cells—condition A) previous peroxide stress. Following this, cells aged for 24 h in water were harvested by centrifugation and resuspended in the same volume of water without (W/O) or with 10% p/v trehalose (T) and incubated at 37 °C/160 rpm for at least another 24 h.

### 2.3. Quantification of hSod1 Inclusions

An aliquot of 2 mL of yeast culture containing 1 mg of cells/mL was centrifuged, washed, and resuspended in 100 μL of distilled water. Next, 2 μL of this cell suspension and 2 μL of n-propyl gallate were mixed in a slide and analyzed by fluorescence microscopy (Olympus IX73 microscope, Tokyo, Japan, DP73 camera, set at 515 nm for excitation and 528 nm for emission), with 100× objective and cellSens Standard software [9]. At least 100 cells per condition were quantified. 

### 2.4. hSOD1 Activity

Soluble proteins from crude extracts were obtained from 50 mg (dry weight) of cells disrupted with 1.5 g of glass beads in sodium phosphate buffer at pH 7.8 and a protease inhibitor mixture (cOmplete, mini, EDTA-free protease mixture inhibitor cocktail, Sigma-Aldrich, Waltham, MA, USA). The protein concentration was determined using the Stickland method. Protein samples (60 μg) were applied in native polyacrylamide gels, which were run at 200 V for around 2 h. Sod1 activity was based on the ability of the enzyme to inhibit a reduction in nitroblue tetrazolium (NBT) by superoxide radicals produced during the photo-oxidation of N,N,N′,N′-Tetramethylethylenediamine (TEMED) by riboflavin. Enzyme activity was quantified by the digital image analysis of achromatic bands considering the area density (arbitrary units) using ImageJ software.

### 2.5. Cell Longevity

Control (glucose growing) and aged cells were plated on a solid dropout 2% glucose complete medium and incubated at 28 °C for 72 h. The number of colonies was counted with the longevity determinate as the ratio between the number of colonies after chronological aging (stress) and glucose-growing cultures (control situation).

### 2.6. Intracellular Oxidation

In total, 50 mg of cells (dry weight) were treated with 10 µM 2′,7′-dichlorofluorescin diacetate (DCFDA, Sigma-Aldrich Merck, Waltham, MA, USA) at 28 °C (control cells) or 37 °C (aged cells) for 30 min. Cells were washed twice with sterile distilled water and disrupted with 1.5 g of glass beads in cold distilled water. Soluble extracts obtained after centrifugation in microcentrifuge were diluted 1:6 in cold water and transferred to black 96-well plates. The fluorescence intensity was determined using the microplate reader SpectraMax M2 at excitation 504 nm and emission 524 nm conditions. The intracellular oxidation was defined as the ratio between the fluorescence of stressed (aging) and non-stressed (control) cells.

## 3. Results and Discussion

### 3.1. Treatment with Trehalose Decreased hSod1 Inclusions

Toxicity associated with misfolding, mislocalization, and protein aggregation are major hypotheses for neurodegeneration in both sporadic and familial ALS. The inclusions contain different proteins, some of which may have an intrinsic tendency to aggregate following genetic mutations, such as Sod1, whereas TDP-43 is found to be aggregated in the motor neurons of the majority of sALS patients [21]. For years, it was believed that only mutated forms of Sod1 could aggregate, but recently, inclusions of WT Sod1 showing post-translational modification associated with damage, such as glycation, have been observed as well [22]. Strikingly, the authors also observed that glycated human WT Sod1 induced the transport of TDP-43 from the nucleus to the cytoplasm, where it undergoes accumulation.

**Figure 1:**
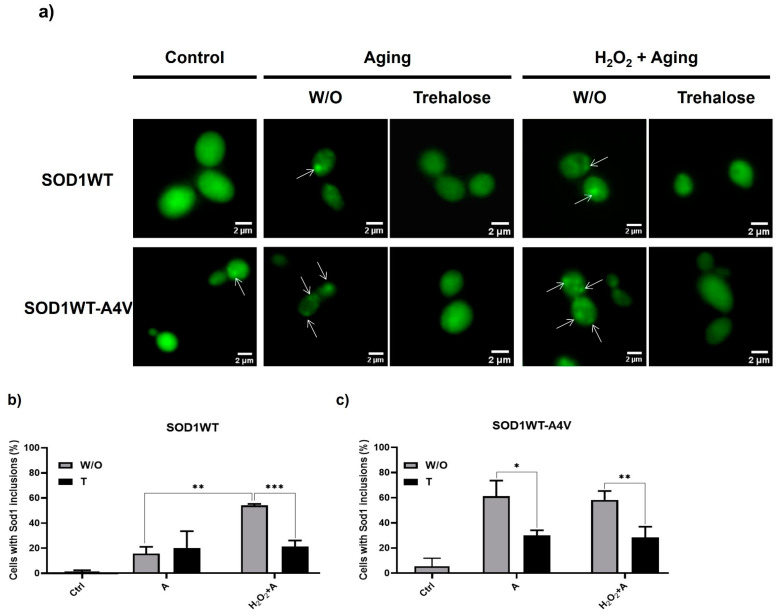
Cells treated with trehalose before oxidative stress showed a decrease in Sod1 inclusions. Yeast cells expressing SOD1WT or SOD1WT-A4V attached to the BiFC system (bimolecular fluorescence complementation) were harvested when growing exponentially on glucose at 28 °C (control condition = Ctrl). Next, they were submitted to oxidative stress through incubation in water (gray bar) or in an aqueous solution of 10% trehalose *w*/*v* (black bars) at 37 °C for 24 h (A = aging-like condition). Cells were also previously treated with 0.4 mM of H_2_O_2_ at 28 °C for 1 h before being transferred to water without (W/O) or with trehalose (T) at 37 °C/24 h (H_2_O_2_ + A condition). (**a**) Representative images of yeast cells expressing SOD1-BiFC. Some SOD1 inclusions are pointed out by white arrows. Quantification of SOD1WT (**b**) or SODWT-A4V (**c**) inclusions and Sod1 levels in yeast cells aged in water (W/O) or in trehalose (T). The results are shown as the mean ± SD of at least three independent experiments. One-way ANOVA with Tukey’s test was used for statistical analysis to compare the difference between the W/O and trehalose condition (significant differences * *p* < 0.05, ** *p* < 0.01, *** *p* < 0.001).

Initially, we established the conditions to induce the oligomerization of the WT hSod1 homodimer as well as the WT-A4V hSod1 heterodimer and investigated the capacity of trehalose to reduce Sod1 inclusions induced by aging, as previously observed [9]. We chose to use this heterodimer because the disease has an autosomal-dominant inheritance pattern, and among Sod1-linked fALS, A4V is very aggressive [23]. Furthermore, heterodimers were found to be more toxic than homodimers [9]. Yeast cells, used in this work, are an excellent model for investigating the effect of oxidative stress, characteristic of the aging process, on the molecular mechanisms of neurodegenerative diseases [24]. Among the main reasons for the choice of this model are the similarity between mammalian and yeast antioxidant responses, the increased number of human proteins related to these diseases with yeast orthologues (such as Sod1), and the capacity of yeast cells to obtain energy exclusively by fermentation when growing on glucose, even in the presence of oxygen. Consequently, yeast cells that are fermenting produce low levels of reactive oxygen species (ROS). To observe the effect of oxidative stress on Sod1 proteinopathy, glucose-growing cells were transferred to water and incubated at 37 °C/160 rpm to increase ROS levels. The elevated temperature with aeration (shaking culture flasks 4/5 full of air) induces severe oxidative stress, which accelerates aging [9,20]. This experimental strategy has been extensively used to study how long a yeast cell stays alive (chronological aging). Part of the culture was previously exposed to peroxide to increase the level of oxidative stress and, consequently, Sod1 oligomerization. As observed in Figure 1a,b, low proportions of aged SOD1WT cells contained WT hSod1 inclusions (less than 20%). However, when the cells were pre-treated with 0.4 mM of hydrogen peroxide solution for 1 h at 28 °C and then subjected to the aging condition, the percentage of cells with WT hSod1 inclusions doubled. As can be seen in Figure 1c, the aging condition without pre-treatment with peroxide produced a high proportion of cells with hSOD1WT-A4V inclusions (~60%). The presence of a Sod1 monomer containing the A4V mutation, which destabilizes the structure, would be enough to misfold the Sod1 dimer and induce oligomerization during the aging process [3,9].

Once the conditions that better induced the formation of SOD1WT and SOD1WT-A4V inclusions were established, the next step was to analyze the effect of trehalose as a pre-treatment (Supplementary Material, Figure S1). This condition would be interesting to verify if trehalose is used as a therapy to retard or avoid ELA symptoms in individuals who know that they may develop ALS because they were diagnosed with the familial form of the disease. Thus, these patients could be treated with trehalose before the appearance of the first symptoms. According to our results, trehalose reduces the percentage of cells with inclusions by approximately 50% in SOD1WT (Figure 1b) and SOD1WT-A4V strains (Figure 1c). This effect was not observed in SOD1WT cells submitted only to cellular aging since, under this condition, the proportion of cells with inclusions was low (<20%). These results show that trehalose supplementation, along with aging, is capable of protecting against Sod1 aggregation. Our results corroborate data from the literature, which verified that trehalose was able to increase the stability of Sod1 preparations [25] and reduce the aggregation of mutant G86R Sod1 in motor neurons of transgenic mice [17]. In this work with the ALS mouse model, trehalose treatment started at the asymptomatic phase of the disease.

**Figure 2:**
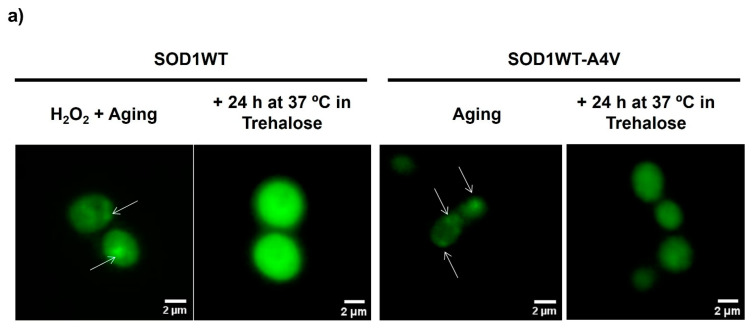

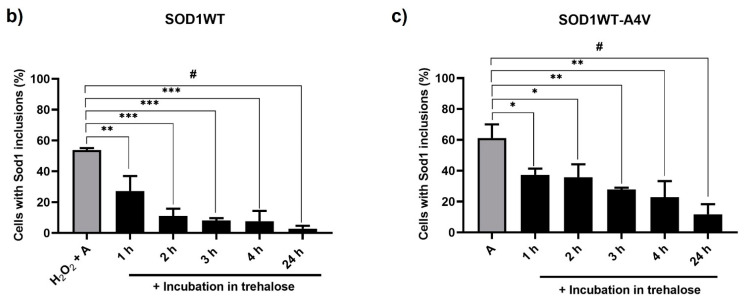
Cells treated with trehalose after 24 h of oxidative stress showed a decrease in Sod1 inclusions. Yeast cells expressing SOD1WT or SOD1WT-A4V attached to the BiFC system (bimolecular fluorescence complementation) were grown on glucose (control condition = Ctrl) before being submitted to oxidative stress through incubation in water (gray bars) at 37 °C for 24 h (A = aging-like condition). For SOD1WT, cells were previously treated with 0.4 mM of H_2_O_2_ at 28 °C for 1 h (H_2_O_2_ + A condition). After 24 h of aging, cells were reinoculated in an aqueous solution of 10% trehalose *w*/*v* (black bars) or maintained in water (white bars) at 37 °C for 24 h. Images were taken before and after 1, 2, 3, 4, and 24 h of incubation in trehalose. (**a**) Representative images of yeast cells expressing SOD1-BiFC after 24 h of reinoculation in trehalose or in water (W/O). Some SOD1 inclusions are pointed out by white arrows. Quantification of SOD1WT (**b**) or SOD1WT-A4V (**c**) inclusions before (A or H_2_O_2_+A) and after cells were reinoculated in trehalose. The results are shown as mean ± SD of at least 3 independent experiments. One-way ANOVA with Tukey’s test was used for statistical analysis to compare the difference between H_2_O_2_ + A (SOD1WT cells-gray bars), (**b**) or A (SOD1WT-A4V cells-gray bars), (**c**) with + incubation at 37 °C in trehalose (significant differences at * *p* < 0.05, ** *p* < 0.01, *** *p* < 0.001, # *p* < 0.0001).

There is currently no known treatment that stops or reverses the progression of ALS. Thus, next, we decided to verify the ability of trehalose to disrupt Sod1 inclusions. This therapy is indicated to treat ALS patients who are diagnosed only after the first symptoms appear. Cells were initially submitted to oxidative stress conditions, which induced Sod1 aggregation, and then were transferred to 10% trehalose solutions for more than 24 h at 37 °C/160 rpm (Supplementary Material, Figure S2). According to Figure 2, a few hours of incubation of both strains with trehalose reduced the proportion of cells with inclusions remarkably, and this decrease was progressive along with the treatment. After 24 h of incubation with trehalose, the percentage of cells showing Sod1 inclusions practically decreased to the levels of the control condition (unstressed cells growing on glucose, Figure 1). Trehalose also disrupted inclusions of WT Sod1 produced by aging and when SOD1WT-A4V cells were doubly stressed (Supplementary Material, Figure S3). Thus, according to our results, trehalose can halt and reverse Sod1 proteopathy, besides reducing the likelihood of the formation of Sod1 inclusions associated with ALS progression.

We observed that the incubation of cells expressing the WT homodimer or mutant heterodimer at 37 °C without trehalose for 48 h reduced the intensity of fluorescence in the cells; therefore, it was not possible to quantify Sod1 inclusions. Since the fluorescence resulted from the interaction of two Sod1, each one linked to half of the Venus fluorescent protein, this suggested that the prolonged incubation of cells at this extreme condition might affect Sod1 dimerization due to Sod1 unfolding. On the other hand, it was observed that the fluorescence intensity of cells gradually increased with trehalose treatment, reinforcing the idea that this sugar avoids Sod1 denaturation and, consequently, agglomeration (Figure 2a). The protective effect of trehalose upon Sod1 seems to be related mainly to the prevention of denaturation, given that aging occurred under non-proliferating conditions, which impair protein expression. It is important to note that cells are not able to proliferate when incubated in a trehalose solution since the other nutrients necessary for growth are absent. However, they might use sugar as a source of energy. We analyzed the intracellular concentration of trehalose before and after aging, and the value remained constant, confirming that trehalose was used to avoid protein denaturation and aggregation.

### 3.2. Treatment with Trehalose Increased Sod1 Activity

One of the main roles of Sod1 is its ability to catalyze the conversion of superoxide radicals into hydrogen peroxide and oxygen. For this, it is necessary that Sod1 be properly dimerized and stable, which is affected by oxidative stress [7,26]. Based on the ability of trehalose to reduce Sod1 inclusions (Figure 1 and Figure 2), next, we verified the effect of this disaccharide on Sod1 activity, which was expressed as the ratio between the activities of stressed (aging with or without trehalose treatment) and non-stressed cells (glucose growing cells at 28 °C).

**Figure 3:**
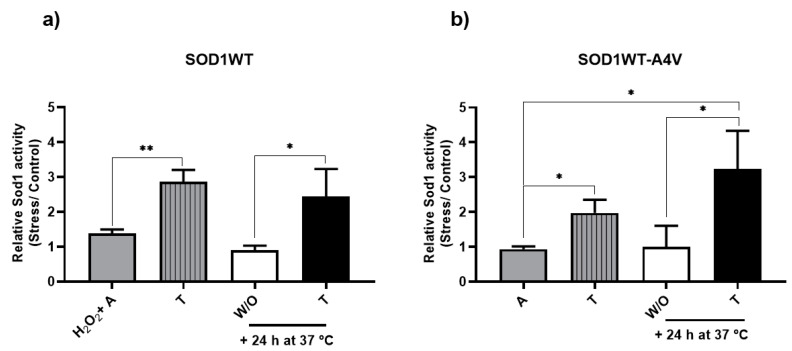
Trehalose increases Sod1 activity. Sod1 activity was analyzed as described in the Methods section and expressed as a ratio between the levels found in aged (stress) and non-stressed (control) cells. Cells were grown on glucose at 28 °C and harvested in the middle of the exponential growth phase (control). (**a**) SOD1WT cells were previously treated with 0.4 mM of H_2_O_2_ at 28 °C for 1 h and incubated at 37 °C for 24 h in water (H_2_O_2_ + A condition, gray bar) or in an aqueous solution of 10% trehalose *w*/*v* (striped, gray bar). After aging (H_2_O_2_ + A condition, gray bar), cells were reinoculated in trehalose (black bars) or maintained in water (W/O, white bar) for more than 24 h at 37 °C. (**b**) SOD1WT-A4V cells were submitted to oxidative stress through incubation in water (A = aging-like condition, gray bar) or trehalose (striped, gray bar) at 37 °C for 24 h. After aging in water (A condition, gray bar), cells were also reinoculated in trehalose (black bars) or maintained in water (W/O, white bar) for more than 24 h at 37 °C. The results are shown as the mean ± SD of at least 3 independent experiments. One-way ANOVA with Tukey’s test was used for statistical analysis to compare the difference between the H_2_O_2_ + A (SOD1WT, gray bar) or A (SOD1WT-A4V, gray bar) condition with aging in trehalose (striped, gray bar), + 24 h at 37 °C in W/O (white bar) and + 24 h at 37 °C for the condition of trehalose (black bar) (significant differences at * *p* < 0.05, ** *p* < 0.01).

When aging was performed in trehalose, the activity of both SOD1WT and SOD1WT-A4V cells increased two-fold (stripped gray bars, Figure 3a and Figure 3b, respectively). A parallel can be drawn between the result of Sod1 activity and the proportion of cells with WT Sod1 or WT-A4V Sod1 inclusions (Figure 1). This suggests that the Sod1 monomers (both WT and mutant) were stabilized, enabling the enzyme to stay active during stress conditions.

When aged cells were treated with trehalose for another 24 h (Figure 3, black bars), the result was similar to the condition in which cells were aged in the presence of trehalose. The activity of SOD1WT cells increased 2-fold in relation to the same condition in water (Figure 3a, white bar) but without presenting a statistical difference with the control condition (Figure 3a, gray bar). This suggests that trehalose can restore Sod1 functionality, even if added after a certain level of damage. After 24 h of aging, the percentage of cells with Sod1 inclusions increased; trehalose added at this point was able to reduce Sod1 oligomerization and restore activity. Heterodimer activity also increased in the presence of trehalose (Figure 3b, black bar), which was statistically different from the control condition (Figure 3b, gray bar). According to our results, the addition of trehalose can disaggregate and avoid the aggregation of the WT-A4V heterodimer, increasing enzymatic activity (Figure 3b). Decreased Sod1 activity can result in increased oxidative stress, the accumulation of ROS, and the induction of oxidative damage to cells. A variety of *SOD1* mutations found in aggressive forms of fALS cause expressive reductions in Sod1 activity, suggesting that Sod1 deficiency may underlie the susceptibility of motor neurons that express mutant Sod1 to injury [10]. In humans, the motor system, contrary to non-neuronal organs, is exceptionally vulnerable to the absence of the ubiquitously expressed Sod1 [27]. Although red blood cells and post-mortem frontal cortex samples of sporadic ALS patients showed normal levels of Sod1 activity, activity was not analyzed in regions exhibiting severe motor neuron degeneration. We observed that the aging process impacted the activity of the heterodimer more than WT Sod1, although the WT form was submitted to a more severe condition (H_2_O_2_ + A) than the mutant. Trehalose was able to increase Sod1 activity of both forms during aging, suggesting that this disaccharide acts as a chaperone, avoiding Sod1 denaturation and oligomerization, which has a great potential to protect against neurodegeneration.

### 3.3. Trehalose Reduced Intracellular Oxidation and Increased Longevity

So far, the results showed the direct action of trehalose on the Sod1, reducing aggregation and increasing its enzymatic activity under conditions of oxidative stress. To verify if the improvement of Sod1 stability conferred by trehalose might help in retaining cellular integrity, we analyzed the levels of intracellular oxidation and cell longevity. The capacity of trehalose to protect against oxidative damage has been known for quite a long time [28,29] and is supported by new data [30].

**Figure 4:**
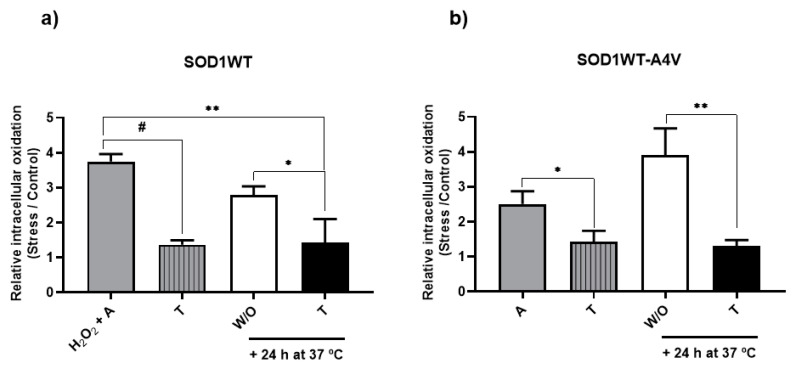
Trehalose decreases the levels of intracellular oxidation. Intracellular oxidation for cells expressing SOD1WT or SOD1WT-A4V was determined usingthe 2′,7′-dichlorofluorescin diacetate probe. Relative intracellular oxidation was defined as the ratio between stressed (aged) and control cells (cells growing on glucose at 28 °C). (**a**) SOD1WT cells were previously treated with 0.4 mM H_2_O_2_ at 28 °C for 1 h and incubated at 37 °C for 24 h in water (H_2_O_2_ + A, gray bar) or in an aqueous solution of 10% trehalose *w*/*v* (striped, gray bar). After 24 h of aging (H_2_O_2_ + A condition, gray bar), cells were also reinoculated in trehalose (black bars) or maintained in water (white bar) for more than 24 h at 37 °C. (**b**) SOD1WT-A4V cells were submitted to oxidative stress through incubation in water (A, gray bar) or trehalose (striped, gray bar) at 37 °C for 24 h. After aging in water (A condition, gray bar), cells were also reinoculated in trehalose (black bars) or maintained in water (white bar) for more than 24 h at 37 °C. The results are shown as the mean ± SD of at least 3 independent experiments. One-way ANOVA with Tukey’s test was used for statistical analysis to compare the difference between the H_2_O_2_ + A (SOD1WT, gray bar) or A (SOD1WT-A4V, gray bar) condition with aging in trehalose (striped, gray bar), +24 h at 37 °C in W/O (white bar) and +24 h at 37 °C for the condition of trehalose (black bar) (significant differences at * *p* < 0.05, ** *p* < 0.01, # *p* < 0.0001).

Figure 4 shows that when cells were aged in trehalose (Figure 4, stripe gray bars), the relative levels of intracellular oxidation were approximately two times lower than without (Figure 4, gray bar). A similar effect was observed in SOD1WT cells treated with trehalose after 24 h of aging in water, where the relative levels of intracellular oxidation in trehalose (black bars) were lower than after 24 h and 48 h of aging without trehalose (Figure 4a), which is in accordance with the Sod1 activities observed in the same conditions (Figure 3a). It was expected that an increase in Sod1 activity would lead to a higher consumption of ROS, decreasing the levels of intracellular oxidation. Trehalose also reduced the levels of the intracellular oxidation of aged SOD1WT-A4V cells (Figure 4b).

**Figure 5:**
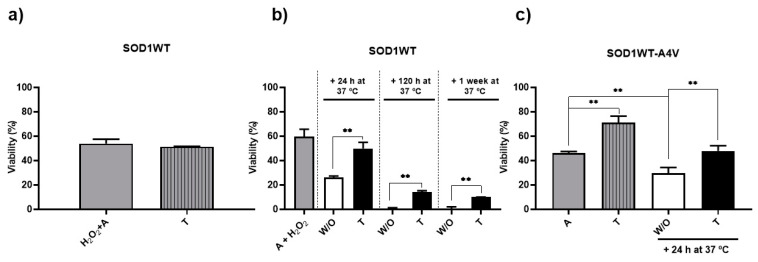
The impact of trehalose on the longevity of the ALS cell model. The viability of SOD1WT or SOD1WT-A4V cells was determined by plating cells on solid glucose media, as described in the Methods section, and expressed as the percentage of colony-forming units (stress/control condition). Both strains were harvested on the mid-log phase growth of glucose at 28 °C (control condition) before they were aged (stress, incubation in water at 37 °C). (**a**) SOD1WT cells were previously treated with 0.4 mM of H_2_O_2_ at 28 °C for 1 h before aging in water for 24 h (H_2_O_2_ + A, gray bar) or in an aqueous solution of 10% trehalose *w*/*v* (striped, gray bar). The results are shown as the mean ± SD of at least 3 independent experiments, and non-statistical differences were found using Student’s *t*-test. (**b**) SOD1WT cells were aged as in (**a**) (gray bar) and reinoculated in trehalose (black bar) or maintained in water (white bar) at 37 °C for more than 24 h, 120 h, and 1 week. The results are shown as the mean ± SD of at least 3 independent experiments. Statistical differences between the W/O (white bar) and T (black bar) condition in each condition were determined using Student’s *t*-test (significant difference ** *p* < 0.01). (**c**) SOD1WT-A4V cells were submitted to oxidative stress through incubation in water (A, gray bar) or trehalose (striped, gray bar) at 37 °C for 24 h. After aging in water (A condition, gray bar), cells were also reinoculated in trehalose (black bars) or maintained in water (white bar) for more than 24 h at 37 °C. The results are shown as the mean ± SD of at least 3 independent experiments. One-way ANOVA with Tukey’s test was used for statistical analysis to compare the differences between the H_2_O_2_ + A (SOD1WT, gray bar) or A (SOD1WT-A4V, gray bar) condition with aging in trehalose (striped, gray bar), + 24 h at 37 °C in W/O (white bar) and +24 h at 37 °C for the condition of trehalose (black bar) (significant differences at ** *p* < 0.01).

The effect of trehalose on cell longevity was initially more expressive in SOD1WT-A4V cells (Figure 5c). When the cells were submitted to cellular aging in trehalose, viability increased approximately twice as much as in water (Figure 5c, gray and stripe gray bars, respectively). A similar effect was observed when the cells were kept in trehalose for another 24 h at 37 °C (Figure 5c, black bar). While the addition of trehalose maintained cell viability, the survival rates of cells kept for another 24 h only in water fell by almost half. These results suggest a direct relationship between the decrease in SOD1WT-A4V inclusions, the increase in SOD1 activity, and increased or maintained cell longevity.

According to Figure 5a, the viability of SOD1WT cells aged in the presence of trehalose did not increase despite the fact that sugar reduced the percentage of cells showing WT Sod1 inclusions from 60 to around 20% (Figure 1b). Despite the high proportion of cells showing Sod1 inclusions, the viability of SOD1WT cells after 24 h in water was around 60% (Figure 5a, gray bar), indicating that cells cope better with inclusions of the WT than the mutant form of Sod1. Thus, trehalose did not increase the survival rates of cells expressing WT Sod1, probably because of the high rates even when aging occurred without the addition of the disaccharide. To investigate the possible effect of trehalose on the viability of the SOD1WT strain, cells were previously stressed in water (aged at 37 °C, 24 h and later treated with H_2_O_2_ 0.4 mM, for 1 h, at 37 °C—condition A+ H_2_O_2_.), and then kept in water or 10% trehalose for up to one week. As can be seen in Figure 5b, although there was a considerable drop in cell viability over time, the survival rates of cells in trehalose were higher than the values for cells in water. While trehalose was added after 24 h of aging and maintained the survival rates at around 60% after 48 h, only 20% of cells incubated in water survived. After 5 days of aging, non-treated cells did not survive, but 15% of cells treated with the sugar were still alive. These survival rates were maintained for one week. These results suggest that trehalose can keep more cells viable for longer.

## 4. Final Considerations

Trehalose is generally recognized as a safe substance and a widely used FDA-approved ingredient in pharmaceutical preparations, including parenteral products. Its safety can facilitate further clinical testing. Furthermore, trehalose is much less expensive than any other ALS therapy available: one kilo of this sugar costs USD 25. According to our results, trehalose is a potent stabilizer of Sod1, increasing its activity. The sugar also acted as a chaperone upon Sod1: it reduced Sod1 aggregation induced by oxidative stress, which is characteristic of the aging process. Sod1 is an important enzyme of the antioxidant system and, consequently, ages in the presence of trehalose, which stabilizes Sod1, decreases the levels of intracellular oxidation, and increases longevity. It appears that trehalose may elicit more significant effects upon cell survival when administered at the presymptomatic stages of the familial form of ALS linked to Sod1WT-A4V, i.e., before the appearance of proteopathy. On the other hand, trehalose significantly extends the life span of cells expressing WT Sod1, as found in the sporadic form of the disease. Besides being able to prevent proteopathy, trehalose disaggregates Sod1 after the induction of proteopathy. Sod1 is very important to motor neurons’ health. The stabilization of its functionality has great potential to retard neurodegeneration. Although our work has made relevant contributions to the potential of trehalose to protect against Sod1 proteopathy, a central mechanism of ALS disease, future tests on more complex ALS models are necessary. Besides the timing of treatment, attaining information on the proper dosing and administration route of trehalose is also necessary in more complex models. It is still unknown if the neuroprotective effect of trehalose follows a dose-dependent pattern. Furthermore, while the intravenous route provides a significantly higher bioavailability of trehalose compared to the oral route, it remains elusive as to whether the gut microbiota mediates the main role of the neuroprotective effect of trehalose.

## Data Availability

The data that support the findings of this study are available from the corresponding author upon reasonable request.

## Supplementary Materials

The following supporting information can be downloaded at https://www.mdpi.com/article/10.3390/antiox13070807/s1. Figure S1: Experimental scheme of treatment with trehalose during chronological aging; Figure S2: Experimental scheme of treatment with trehalose after chronological aging; Figure S3. SOD1WT-A4V cells treated with trehalose after oxidative stress showed a decrease in SOD1 inclusions. Table S1: Number of viable cells used to obtain the survival rates.
