# Supplementary material for: Trehalose Protects against Superoxide Dismutase 1 Proteinopathy in an Amyotrophic Lateral Sclerosis Model
Source: Antioxidants (Basel). 2024 Jul 3;13(7):807. doi: 10.3390/antiox13070807 (PMC11274086; doi:10.3390/antiox13070807)
Supplement: Supplementary file 1 [file antioxidants-13-00807-s001.zip › antioxidants-3066456-supplementary.pdf]

Supplementary Figure S1

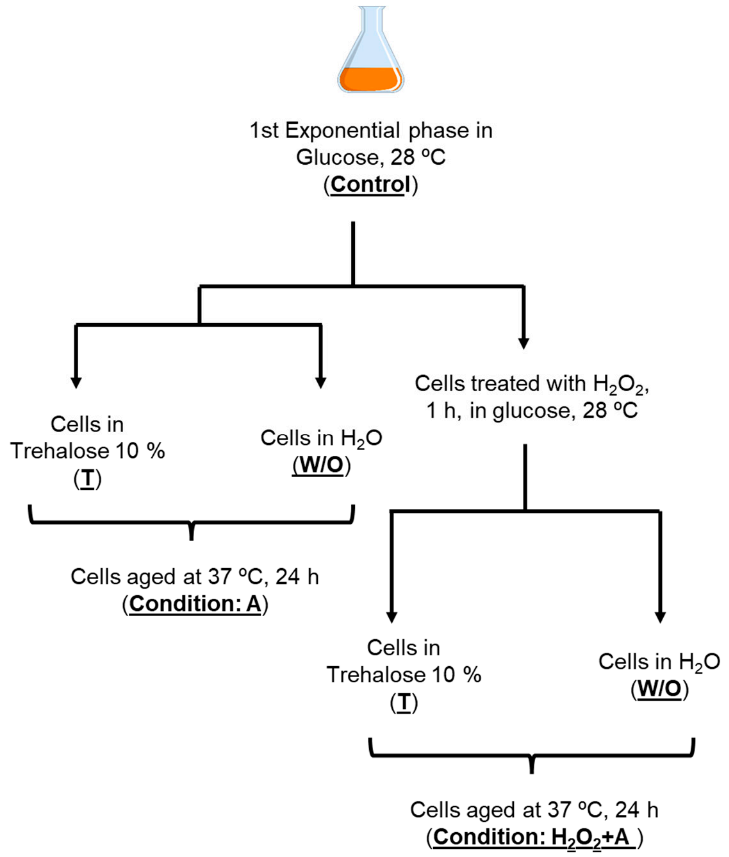

Figure S1. Experimental scheme of treatment with trehalose during chronological aging. Condition A: mid-exponential phase cells (control) were centrifuged at 5000 rpm/5 min, washed twice with sterile distilled water, and resuspended in the same volume of water or trehalose at 10 %. Cells were incubated at 37 °C/160 rpm for 24 h (aged cells). Condition H_2O_2 + A: mid-exponential phase cells (control) were also treated with H_2O_2 at 0.4 mM in glucose, at 28 °C / 160 rpm for 1 h, before chronological aging, as described above.

Supplementary Figure S2

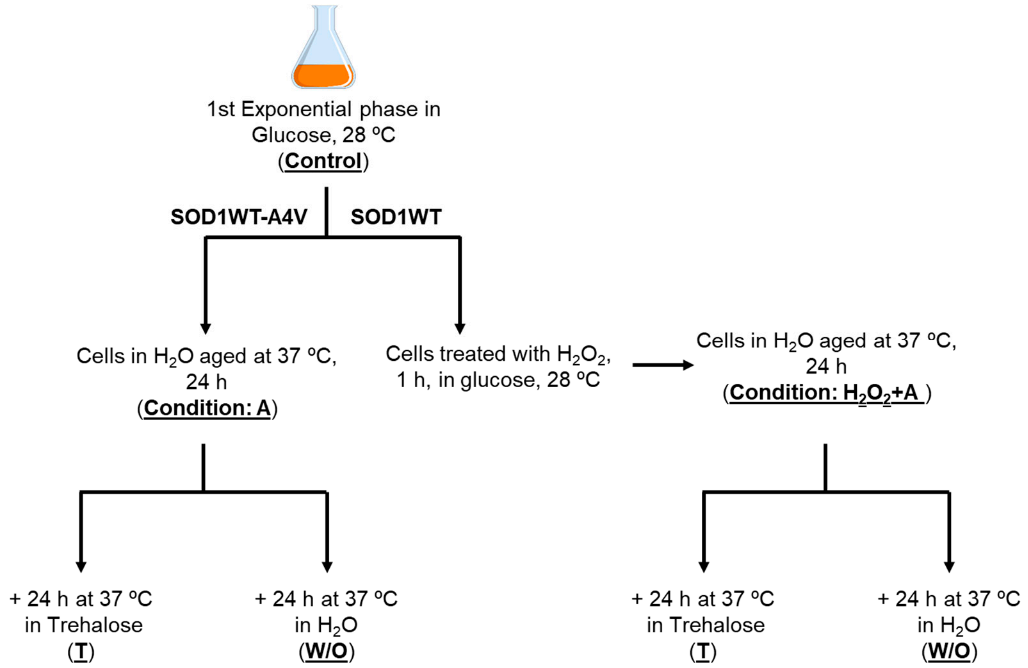

Figure S2. Experimental scheme of treatment with trehalose after chronological aging. SOD1WT-A4V cells: mid-exponential phase cells (control) were centrifuged at 5000 rpm/5 min, washed twice with sterile distilled water, resuspended in the same volume of water, and incubated at 37 °C/160 rpm for 24 h (aged cells). Aged cells were centrifuged at 5000 rpm/5 min, resuspended in the same volume of trehalose at 10 %, and incubated at 37 °C/160 rpm for 24 h. SOD1WT cells: to induce the formation of SOD1WT inclusion, mid-exponential phase cells (control) were treated with H_2O_2 at 0.4 mM in glucose at 28 °C / 160 rpm for 1 h. Treated cells were centrifuged at 5000 rpm/5 min, washed twice with sterile distilled water, resuspended in the same volume of water, and incubated at 37 °C/160 rpm for 24 h (aged cells). Aged cells were centrifuged at 5000 rpm/5 min, resuspended in the same volume of trehalose at 10 %, and incubated at 37 °C/160 rpm for 24 h.

Supplementary Figure S3

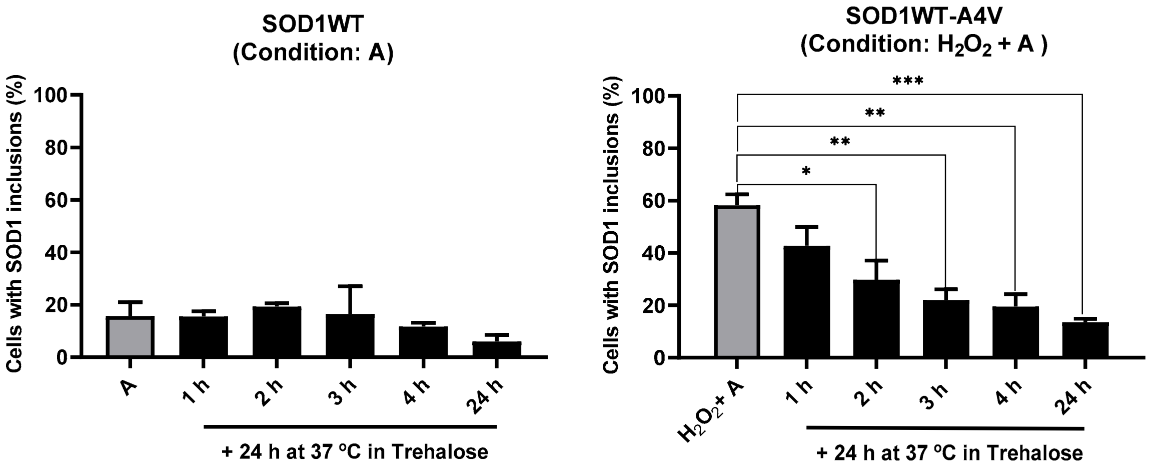

Figure S3. SOD1WT-A4V cells treated with trehalose after oxidative stress showed a decrease in SOD1 inclusions. Quantification of cells with SOD1 inclusion. SOD1WT or SOD1WT-A4V cells were submitted to oxidative stress through incubation in water (gray bar) at 37 ºC for 24 hours (A = aging-like condition). For SOD1WT-A4V, cells were previously treated with 0.4 mM of H_2O_2 at 28 ºC for 1 hour (H_2O_2 + A condition, gray bar). After aging, cells were reinoculated in trehalose (black bars) or maintained in water (white bars) at 37 ºC for 24 hours. Images were taken in the first 4 hours and after 24 hours of incubation. One-way ANOVA with Tukey's test was used for statistical analysis to compare the difference between H_2O_2 + A (SOD1WT-A4V cells- gray bars) or A (SOD1WT cells-gray bars) with + 24 hours at 37 ºC in trehalose (significant differences at * $p<0.05$, ** $p< 0.01$, *** $p< 0.001$).

Table S1–Number of viable cells used to obtain the survival rates.

The tables below show the average number of viable cells under each condition for the SOD1WT and SOD1WT-A4V strains. The results are shown as the mean of at least three independent experiments (biological replicates), and at least three plates were used in each experiment (technical replicates).

SOD1WT strain

a) Trehalose before aging.

**Condition**	**Number of viable cells (X 10^9). Cells that proliferated in solid media and generated a visible colony**
Control	154
H_2O_2 + A	74
T	80

b) Trehalose after aging.

**Condition**	**Number of viable cells (X 10^9). Cells that proliferated in solid media and generated a visible colony**
Control	154
A+ H_2O_2	92
W/O 24 h	40
T 24 h	77
W/O 120 h	2
T 120 h	22
W/O 1 week	0
T 1 week	15

SOD1WT-A4V strain.

**Condition**	**Number of viable cells (X 10^9). Cells that proliferated in solid media and generated a visible colony**
Control	233
Aging	108
T	165
W/O (+ 24 h at 37 ºC)	69
T (+ 24 h at 37 ºC)	112
